# Generation of a PAX6 knockout glioblastoma cell line with changes in cell cycle distribution and sensitivity to oxidative stress

**DOI:** 10.1186/s12885-018-4394-6

**Published:** 2018-05-02

**Authors:** Beate Hegge, Eva Sjøttem, Ingvild Mikkola

**Affiliations:** 1 Department of Pharmacy, Research Group of Pharmacology, University of Tromsø – The Arctic University of Norway, 9037 Tromsø, Norway; 2 Department of Medical Biology, Molecular Cancer Research Group, University of Tromsø – The Arctic University of Norway, 9037 Tromsø, Norway

**Keywords:** PAX6, U251, Glioblastoma, Cell cycle, Oxidative stress, CRISPR-Cas9, Migration, Proliferation, Morphology, Colony-formation

## Abstract

**Background:**

The transcription factor PAX6 is expressed in various cancers. In anaplastic astrocytic glioma, PAX6 expression is inversely related to tumor grade, resulting in low PAX6 expression in Glioblastoma, the highest-grade astrocytic glioma. The aim of the present study was to develop a PAX6 knock out cell line as a tool for molecular studies of the roles PAX6 have in attenuating glioblastoma tumor progression.

**Methods:**

The CRISPR-Cas9 technique was used to knock out PAX6 in U251 N cells. Viral transduction of a doxycycline inducible EGFP-PAX6 expression vector was used to re-introduce (rescue) PAX6 expression in the PAX6 knock out cells. The knock out and rescued cells were rigorously characterized by analyzing morphology, proliferation, colony forming abilities and responses to oxidative stress and chemotherapeutic agents.

**Results:**

The knock out cells had increased proliferation and colony forming abilities compared to wild type cells, consistent with clinical observations indicating that PAX6 functions as a tumor-suppressor. Cell cycle distribution and sensitivity to H_2_O_2_ induced oxidative stress were further studied, as well as the effect of different chemotherapeutic agents. For the PAX6 knock out cells, the percentage of cells in G2/M phase increased compared to PAX6 control cells, indicating that PAX6 keeps U251 N cells in the G1 phase of the cell cycle. Interestingly, PAX6 knock out cells were more resilient to H_2_O_2_ induced oxidative stress than wild type cells. Chemotherapy treatment is known to generate oxidative stress, hence the effect of several chemotherapeutic agents were tested. We discovered interesting differences in the sensitivity to chemotherapeutic drugs (Temozolomide, Withaferin A and Sulforaphane) between the PAX6 expressing and non-expressing cells.

**Conclusions:**

The U251 N PAX6 knock out cell lines generated can be used as a tool to study the molecular functions and mechanisms of PAX6 as a tumor suppressor with regard to tumor progression and treatment of glioblastoma.

**Electronic supplementary material:**

The online version of this article (10.1186/s12885-018-4394-6) contains supplementary material, which is available to authorized users.

## Background

Malignant gliomas are tumors of glial cell origin. The most common and aggressive brain tumor in adults is Glioblastoma (GBM), consisting of cells with astrocytic features. GBM can arise either as a primary tumor, or from anaplastic astrocytoma (AA). While AAs are grade III tumors according to World Health Organization (WHO) [1], GBM is grade IV, the highest-grade astrocytoma [2], defined by uncontrolled proliferation, infiltration, proneness for necrosis, sturdy angiogenesis and genomic instability [3]. Surgical cure is nearly impossible due to early invasion into the central nervous system. Only 10% of patients getting surgical treatment combined with radiotherapy and chemotherapy survive 5 years after diagnosis [4, 5]. Chemotherapeutic treatment options are few and efficacy poor for glioma patients. In addition, chemotherapy resistance is common [6]. Overall, patient survival is approximately 12 months from diagnosis [7, 8]. For reasons unknown, incidence of GBM is rising, and there has been very little improvement in clinical outcomes for GBM patients over the last decade [9]. Increased knowledge about the genetics of GBM is crucial for developing new, targeted therapy.

PAX6 is a transcription factor involved in embryonic brain development, and is also present in the adult brain [10–12]. It has been shown that PAX6 is expressed in a number of different cancer cell lines, and that it can act as a tumor suppressor or in an oncogenic manner, depending on the tissue affected [13–16]. In gliomas, PAX6 acts as a tumor suppressor reducing tumor growth [16], and PAX6 expression is reduced with the malignancy of glioma [17, 18]. PAX6 is used as a prognostic marker for malignant astrocytic gliomas, where low levels of PAX6 expression in AA and GBM correlates with unfavorable patient outcomes [18]. GBM expresses lower levels of PAX6 compared to adjacent healthy tissue, and AAs typically have three folds more PAX6 expression compared to GBM [18]. There has not been identified PAX6 mutations in gliomas, and the lower expression is probably caused by epigenetic changes as the tumors develop [19].

PAX6 is found to suppress cell proliferation, cell invasiveness, and colony formation. In glioblastoma cell lines, ectopic PAX6 expression downregulates matrix metalloproteinase-2 (MMP2) and suppresses invasiveness [20]. PAX6 is also involved in inhibiting angiogenesis in gliomas, as it is found to downregulate expression of vascular endothelial growth factor A (VEGFA) [21], the main angiogenic factor overexpressed in GBM. PAX6 has been showed to inhibit WNT5A-mediated glioblastoma stem cell (GSC) differentiation into endothelial-like cells, where silencing of PAX6 by activation of the AKT- pathway increased proliferation, vascularization, and invasive growth [22]. There are also strong indications that PAX6 is involved in regulating glioblastoma cell cycle by arresting cells in G0/G1-phase, leading to slower proliferation [17]. Chang and colleagues showed that U251 glioblastoma cells are more sensitive to oxidative stress when overexpressing PAX6 [3, 23]. The common chemotherapeutic treatment option Temozolomide (TMZ) has been shown to increase PAX6 expression, and to depend on PAX6 to function [24]. However, the mechanisms behind the tumor suppressor functions of PAX6 in glioblastoma are not fully revealed, and further studies are needed both for the development of new drugs, and for the understanding of the observed resistance to present glioblastoma drug treatments.

In the present study, we have used the CRISPR-Cas9 technology to knock out PAX6 in a commonly used cell line for GBM research, namely U251. We have investigated the morphologies of our knock out (KO) cells and have shown that the removal of PAX6 causes increased proliferation, migration and colony forming abilities. Furthermore, we have observed a shift in the cell cycle distribution of the PAX6 KO cells compared to WT cells. Interestingly, we also found that PAX6 KO cells are more resilient than WT cells to oxidative stress. This was confirmed by reintroducing PAX6 into the KO cells by retroviral transduction.

## Methods

### Cell culture and treatment

The U251 N cell line was a kind gift from Dr. Hrvoje Miletic, University of Bergen, Norway [25]. The U251 N cells, the CRISPR-Cas9 cells generated from U251 N (this paper), and the HEK Phoenix cells (ATTC #CRL-3213) were all cultured in Dulbecco’s Modified Eagle’s Medium - high glucose (cat#D5796 Sigma) supplemented with 10% FCS (Biochrom AG, Berlin, Germany) and 1% penicillin/streptomycin (cat#P0781, Sigma). Cells were transfected using Lipofectamine 2000 (cat#11668–019, Invitrogen, Calsbad, CA). Authentication of the original U251 N cell line and generated cells, were performed at the accredited (ISO/IEC 17025) Centre of Forensic Genetics, University of Tromsø – The Arctic University of Norway. No ethics approval and informed consent were required to use any of the above mentioned cell lines in this study.

### Generation of PAX6 KO cell-lines by CRISPR-Cas9 genome editing

Oligos for guide RNAs were selected with the help of an online CRISPR-Cas9 Design tool, crispr.mit.edu/. We chose three of the guide RNA sequences suggested by the program and added 5’CACCG-3′ to the five prime end of the oligo. Oligos for guide RNAs are displayed in Table 1.

**Table 1:** Guide RNA sequences

Guide 1	Forward	5’-CACCGTGTCAACGGGCGGCCACTGC-3′
	Reverse	5′- AAACGCAGTGGCCGCCCGTTGACAC-3′
Guide 2	Forward	5’-CACCGAGCGGAGTGAATCAGCTCGG-3′
	Reverse	5′- AAACCCGAGCTGATTCACTCCGCTC-3’
Guide 3	Forward	5′- CACCGTGGTGTCTTTGTCAACGGG-3’
	Reverse	5′- AAACCCCGTTGACAAAGACACCAC-3’

The guide RNA sequences chosen had the least number of potential off-target sites calculated by the CRISPR Cas9 Design tool. Annealed guide oligos were cloned into the CRISPR-Cas9 expression vector pSpCas9 (BB)-2A-GFP (PX458) (#48138 Addgene plasmid [26]. The vector was linearized by BbsI (cat#R0539S New England BioLabs), and guide oligos were cloned into the vector by T4 DNA ligase (cat#15224–041, Invitrogen). The three different plasmids were separately transfected into U251 N cells. The next day, single cells were sorted into 96-well plates by FACS. Two weeks later colonies emerged and single colonies were expanded into 6 well plates. PAX6 knockout cells and PAX6 control cells were selected by western blot. Successful knockout was verified by genome sequencing: The N-terminal part of PAX6 was PCR amplified by use of the PAX6 oligos (Table 2). The PCR product was by Zero Blunt PCR cloning kit (Invitrogen cat#44–0302) and transformed into E.coli DH5-alpha. Colonies were sequenced by M13 primers. Four PAX6 positive single cell clones were verified by sequencing and kept as controls. Potential off-target sites were also sequenced. A list of potential off-target sites were generated by the online CRISPR Cas9 Design tool, crispr.mit.edu/. We chose the two potential off target-sites that were located in an exon or splice sites. For guide 2, these sites were located in *NOL6* (Nucleolar Protein 6) and *PPP1R9A* (Protein Phosphatase 1 Regulatory Subunit 9A). Primers used for amplification of the genomic areas are displayed in Table 2, and cloning and sequencing were done as described above The cells were used in experiments for a maximum of five passages after pooling single cells clones, to avoid the potential of one clone dominating the pool.

**Table 2:** Primers for amplification of target- and off-target genomic DNA

Gene	Forward Primer	Reverse Primer
*PAX6*	5’-CTGGTGGTCCTGTTGTCCTT-3′	5’-CAGAATTCGGGAAATGTCG-3′
*NOL6*	5’-ACTTGCTCAACCACCCATTC-3′	5’-ATGAGAGCCTGGATCTGGTG-3′
*PPP1R9A*	5’-CACAAATGTGGAACCCACTG-3′	5’-CTAAAAGCAAAGTTTTACTATTCAGGT-3′

### Generation of doxycycline inducible EGFP-PAX6 rescue cells

Retroviral transduction was used to stably reintroduce PAX6 into knockout cells. PAX6 was cloned into pDest-LRT-EGFP (gift from Trond Lamark, University of Tromsø – The Arctic University of Norway) by gateway cloning (Gateway LR clonase cat#11791–020 BP clonase cat#11789–020, Invitrogen, Calsbad, CA). The resulting plasmid pLRT-EGFP-mPAX6, was transfected into HEK Phoenix cells (ATTC# CRL-3213) producing gag-pol and envelope protein. Supernatant was harvested and sterile filtrated at day 2 and 3 after transfection. 2 ml of 25% supernatant in culture medium and 5 mg/ml proteaminsulfate was added to individual U251 PAX6 KO cell lines. The following day 1 ml supernatant was removed from cells and 3 ml culture medium was added. 24 h later the cells were washed 2 times in culture medium. Infected cells were selected by 10 μg/ml blasticidin (cat#A11113903 GIBCO, Thermo Fischer). Verification of inducible EGFP-PAX6 was done by titration of doxocyclin (Dox) (cat#D3447, Sigma**)** at the concentrations; 10, 25, 50, 100, 250 and 500 ng/ml and visualization by green fluorescence and anti-PAX6 western blot. A concentration of 10 ng/ml Dox was used for experiments if not otherwise indicated in the text. When indicated, EGFP-PAX6 expressing cells were FACS sorted 1 day after stimulation with 100 ng/ml Dox, for use in experiments The cell sorting was done on a FACSAria III from Becton & Dickinson (BD) at the Advanced Microscopy Core Facility (AMCF) at the Faculty of Health Sciences, UiT – The Arctic University of Tromsø, Norway.

### Western blots

Cell lysates were prepared by washing 90% confluent cells in 6 well plates with PBS, detaching and scraping in 100 μl 2xSDS PAGE buffer (100 mM Tris-HCl, pH 6.8, 4% SDS, 20% glycerol, 0.2% bromphenolblue and freshly added DTT to 0.2 M). Cells were boiled at 100 °C for 5 min. Cell lysates were sonicated 2.5 min in a icecold water bath, using the Bioruptor (Diagenode) with settings “High”, 30 s on/off cycles. Samples were briefly centrifuged before being loaded in premade gels (cat# NW04122BOX Invitrogen). See Blue Plus2 Prestained Standard (cat#LC5925 Invitrogen), Super Signal Molecular Weight protein ladder (cat#84785, Thermofisher) and MagicMark XP Western Protein Standard (cat#LC5602 Invitrogen) were used as molecular weight markers. Gels were run 35 min, at 200 V, 120 mAmpere. Proteins were blotted onto Li-Cor Odyssey nitrocellulose membranes (cat #926–31,092). Rabbit anti-PAX6 antibody (cat#AB2237, Millipore) at 1:1000 and mouse anti-Pax6 antibody(Cat# pax6, RRID:AB_528427, Developmental Studies Hybridoma Bank, University of Iowa) at a 1:37 dilution was used for screening single cell clones, and rabbit anti-Actin (cat#A2066, Sigma-Aldrich) at a 1:1000 dilution as a loading control. Anti-rabbit 680LT (cat#926–68,023), anti-rabbit 800CW (cat#926–32,213) or anti-mouse 800 CW (cat#926–32,212) IRDye secondary antibodies (LI-COR Biosciences) were used at a dilution of 1:10000 in TBST. Images were acquired on Odyssey Sa (LI-COR Biosciences).

### Immunocytochemistry

Cells (50000) were seeded at sterile fibronectin coated coverslips in 24 well plates, and left in a cell incubator overnight (ON). The cells were fixed in 4% paraformaldehyde, 20 min at room temperature (RT), washed in 1xPBS and permeabilized by Methanol, 5 min, RT. Cells on coverslips were washed in PBS and blocked using 5% BSA, 20 min, RT. PAX6 primary antibody was added (Cat# pax6, RRID:AB_528427, Developmental Studies Hybridoma Bank, University of Iowa) at a dilution of 1: 37 in PBS, 1% BSA, 1 h, RT. The coverslips were washed in PBS, 1% BSA containing DAPI, and subsequently incubated in secondary antibody (Alexa Fluor® 555-conjugated goat anti-mouse IgG, Cat# A-32727 (1:1000). Life Technologies and Alexa Fluor® 647-conjugated goat anti-rabbit IgG (Cat# A32733 (1:5000)). One hour later the coverslips were washed repeatedly in PBS, 1% BSA and then in H_2_O. The coverslips were attached to object glass using Mowiol mounting media and left to dry ON at RT. The next day the cells were photographed by Zeiss LSM 780 inverted confocal microscope with a 63 × 1.4 plan-Apochromat NA objective at different wavelengths of light to visualize EGFP and secondary antibody tags.

### Colony-forming assay

Cells were seeded in 6 well plates in triplicates of either 200, 500 or 1000 cells/well. The cells were incubated for 12 or 16 days in culture medium. At day 12 or 16, cells were fixed 5 min in 3:1 methanol: acetic acid and then stained 15 min in 0.005% crystal violet. Colonies were counted and the percent of cells forming colonies compared to plated cells (100%) were calculated. Morphology of clones and cells in general, were investigated by use of Zeizz Axiovert S100 microscope and the NIS-Elements Documentation software (Nikon).

### Migration assay

Cells were seeded in Ibdi 2 well silicone inserts (Ibdi cat# 81176) in 24 well plates; 20,000 cells per insert well to obtain 95–100% confluency the next day. To inhibit further cell proliferation, the cells were treated with 50 μg/ml Mitomycin C (cat# M4287, Sigma) in complete culture medium for 2 h. The cells were washed twice with complete medium, and inserts were removed. The cell gaps were photographed (timepoint 0). Cells were returned to the tissue culture incubator and photographed again after 20, 48 and 72 h, at the same positions along the gap. The distance of cell migration was calculated by digitally drawing ten evenly spaced horizontal lines from edge to edge of the wounds at 0 and 20 h using NIS Elements BR 2.3 (Nikon). The mean value was calculated for each scratch. For each experiment, 4–10 scratches were measured per cell type. Three independent experiments were analyzed. To determine the statistical relevance of differences in migration between the different cell lines, Student’s t-test was performed.

### Proliferation assay

Cells were seeded in 6 well plates, 50,000 cells per well, done in triplicate at Day 0, and counted on Day 1, 2 and 3 by use of a hematocytometer. Alternatively, 2200 or 4400 cells were seeded per well in 96 well dishes and analysed by Cell Titer Glo (G7571, Promega) according to the manufacturers instruction. This assay detect ATP, and thus the amount of viable cells in a culture. The luminescence was recorded using a CLARIOstar microplate reader (BMG Labtech).

### Cell cycle assay

Cells at 70–90% confluency were detached by trypsin and washed twice in PBS. PBS was removed, leaving 200 μl for resuspension. While carefully vortexing cells, 2 ml cold 70% EtOH was added dropwise. Cells were incubated at 4 °C for a minimum of 1 h. After washing twice in PBS, cells were resuspended in 200 μl 50 μg/ml Propidium iodide (cat#P4170 Sigma) diluted in dH_2_O. 50 μl 100 μg/μl RNase A (cat#EN0531 Thermo Scientific) was added, and cells were left in the dark, at room temperature for 30 min, before analyzing by flow cytometry in a LSR Fortessa (BD).

### Oxidative stress and apoptosis assay

Cells were seeded to 50% confluency in complete medium. The next day medium was replaced with 300 μM hydrogen peroxide containing medium, and left for 24 h. Medium was collected and cells were washed in PBS that was collected. Cells were trypsin treated, and the previously collected medium was used to inactivate the trypsin. Cells, collected medium and PBS were centrifuged at 1200 rpm, 3 min. Cells were further treated according to protocol of the FITC Annexin V Apoptosis Detection Kit I (cat#556546 BD Pharmigen), and analyzed by flow cytometry at PI and FITC channels in a LSR Fortessa (BD).

### Chemotherapy treatment of cells

At 50% confluency, cell growth medium containing chemotherapeutic agents were added to the cells for a 72 h incubation. DMSO was used as a solvent. The concentrations were as follows: Temozolomide (TMZ) (cat#T2577, Sigma Aldrich) 250 μM, Withaferin A (WA) (cat#W4394, Sigma Aldrich) 1.5 μM, Sulforaphane (SFN) (cat#574215, EMD Millipore) 10 μM. Cells were also incubated in growth medium containing 0.2% DMSO, or in growth medium. Cells were photographed and apoptosis assays were performed as described above.

### RNA extraction and reverse transcriptase-qPCR

RNA purification, cDNA synthesis and qPCR reactions are previously described in [27]. Housekeeping genes for standardizations were TFRC and GAPDH. The primer pairs for the housekeeping genes and for MMP2 were purchased as KiCqStart primers from SIGMA (PrimerPair ID: H_TFRC_1, H_GAPDH_2 and H_MMP2_1). Other primers were designed by use of Primer3 [28]. Primer3 designed primer pairs for qPCR are shown in Table 3.

**Table 3:** qPCR primer pairs

Gene	Forward Primer	Reverse primer
*CAV1*	5´-GAGCTGAGCGAGAAGCAAGT-3′	5´-CAAATGCCGTCAAAACTGTG-3´
CyclinD1	5´-CTGGATGCTGGAGGTCTGCGAG-3´	5´-GCCGTCAGGGGGATGGTCTC-3´
*P27*	5´-CAGCTTGCCCGAGTTCTACT-3´	5´-TGTCCTCAGAGTTAGCCGGA-3´
*NRF2*	5´-GCTTTCAACCAAAACCACCCT-3´	5´-TGATGCCACACTGGGACTTG-3´

## Results

### Establishing PAX6 knockout cells by CRISPR-Cas9 technology

**Fig. 1:**
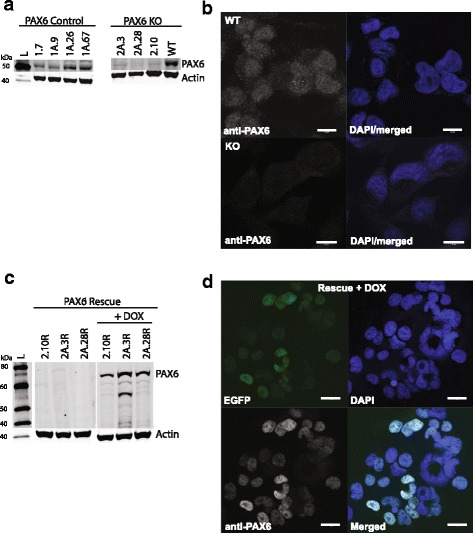
PAX6 is knocked out by CRISPR-Cas9 technology in U251 N glioblastoma cells and reintroduced by an EGFP-PAX6 expressing plasmid (**a**) Western blot on cell lysates originating from single cell clones after CRISPR-Cas9 treatment. PAX6 Control cells have undergone CRISPR-Cas9 treatment and single cell sorting but still express PAX6. Detection by anti-PAX6 antibody. Actin staining was used for loading control. KO, knock out; WT, wild type U251 N; L, Molecular weight marker. **b** Immunocytochemistry (ICC) on WT U251 N and pooled PAX6 KO cell lines (2A.3, 2A.28 and 2.10). Anti-PAX6 antibody detect endogenous PAX6 protein, DAPI staining identifies the nucleus. Scale bars indicate 10 μm. **c** Western blot on the individual PAX6 KO cell lines transduced with a Dox inducible EGFP-PAX6 expressing plasmid (2A.3R, 2A.28R and 2.10R). The right panel show cells treated with 10 ng/ml Dox for 24 h. Anti-PAX6 and anti-Actin antibodies were used for detection. **d** ICC of Dox treated pooled Rescue cells expressing EGFP-PAX6. EGFP is detected as green fluorescence, anti-PAX6 antibody stain PAX6 protein, and the nucleus is stained by DAPI in blue. Scale bar indicates 20 μm. Western blots and ICCs were performed on more than three samples of each cell line and showed the same results

The CRISPR-Cas9 technology was used to knockout PAX6 from the U251 N glioblastoma cell-line. Three different guide RNAs were chosen (crispr.mit.edu/). The guide RNAs were designed to create mutations close to the 5’end of the PAX6 gene, between positions + 25 to + 58 relative to the transcription start site (TSS) (Additional file 1: Figure S1). The PAX6 protein expression in expanded single cell clones were evaluated by western blot using anti-PAX6 antibody. One of the guide RNAs produced several PAX6 knockout (KO) clones. In addition, some of the single cell clones had a marked reduction in PAX6 expression compared to wild type (WT) U251 N cells, indicative of heterozygous knockout of PAX6 (data not shown). Sequencing of PAX6 KO cells showed that cell lines originating from single cell clones had a variety of mutations creating stop codons downstream of the TSS (Additional file 2: Figure S2), therefore it will not be correct to label them as clones and they will hereby be referred to as cell lines or simply cells. We selected three PAX6 KO cell lines obtained by use of guide 2, namely 2.10, 2A.3 and 2A.28. As control cells, we used four single cell clones that had been through the CRISPR-Cas9 protocol of transfection and selection but remained PAX6 positive, namely 1.7, 1A.9, 1A.26 and 1A.67. By performing western blot (Fig. 1a) and immunocytochemistry (ICC) (Fig. 1b) using anti-PAX6 antibody, we verified the absence of PAX6 in the knockout cells. All the PAX6 KO cell lines were further transduced with a retroviral vector containing a Doxycycline (Dox) inducible EGFP-PAX6 gene to create cells that are PAX6 expression “rescued”. We observed a concentration dependent induction of PAX6 expression when Dox concentrations between 10 ng/ml and 500 ng/ml were tested (data not shown). A concentration of 10 ng/ml Dox was sufficient to induce a PAX6 expression similar to that of WT cells (data not shown), and this concentration was therefore used for further experiments with the PAX6 Rescue cells. Figure 1C shows a western blot of the three different PAX6 Rescue cell lines with and without Dox. The uninduced cells do not show EGFP-PAX6 expression (nor PAX6 expression). However it should be noted that for some experiments we observed weak EGFP-PAX6 expression without the addition of Dox, indicating leakiness of the inducible system. Leakiness of inducible expression systems is not an unusual phenomenon [29, 30]. The presence of PAX6 in the Rescue cells were confirmed by comparing ICC of the pooled PAX6 KO cells and pooled PAX6 Rescue cells. In addition to the green fluorescence mediated by the EGFP-tagged PAX6 protein, cells were DAPI stained, and immune-stained by PAX6 antibody (Fig. 1d). The intensity and location of the green fluorescence and the PAX6 immunostaining coincided and thereby confirmed induced expression of the EGFP-PAX6 protein. However, it also showed significant variability in the amount of PAX6 protein expressed in individual cells upon Dox induction. The variability in PAX6 expression amongst cells cannot be detected by western blot. Although it should be considered when performing experiments on the Rescue cells as PAX6 is known to have dose dependent effects.

### Morphological variations amongst the PAX6 KO cell lines

**Fig. 2:**
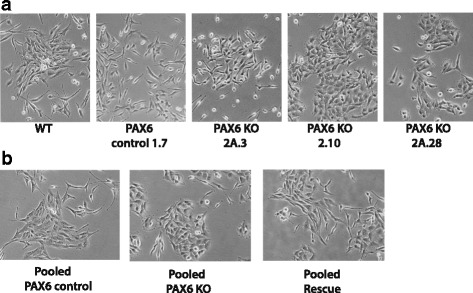
The PAX6 KO cell lines show morphological variations. Cells were micro-photographed at 20×. **a** KO cells and controls. **b** Pooled PAX6 KO-, PAX6 Control, and PAX6 Rescue cells

It was apparent that there were morphological varieties in the PAX6 KO cells compared to the U251 N WT cells (Fig. 2). U251 cells are a mixture of morphological diverse cells that also form different types of colonies [31]. The WT cells consists of elongated neuron-like cells and some shorter cells. The U251 CRISPR-Cas9 PAX6 control 1.7 cells retains morphology as U251 N WT, whereas the PAX6 KO 2.10 cells have shorter triangular cobblestone-like morphology (Fig. 2a). The PAX6 KO cell lines 2A.3 and 2A.28 show similar morphology to WT, with 2A.28 having elements of the cobblestone morphology of the 2.10 cells (Fig. 2a). However, all three KO cell lines consist of varieties of cell shapes and sizes that are all present more or less in U251 N WT. However, each of them have the dominating cell morphologies described above. The three KO cell lines (2.10, 2A.3 and 2A.28) were pooled to diminish potential clonal effects in further experiments. We also pooled four different CRISPR-Cas9 PAX6 control cell lines, amongst these the 1.7 cells. Even though the pooled KO cells consisted of morphological divergent cells, the triangular cobblestone morphology dominated, while the pooled PAX6 control cells had similar morphology to the WT and the 1.7 cells (Fig. 2b) The morphology of the pooled Rescue cell lines are similar to pooled PAX6 KO, but with more dominance of elongated cells (Fig. 2b). The pooled cells were kept for five passages for experiments, with the described variety in morphology being constant during this time. We showed that knocking out PAX6 alters the cell morphology, with the main feature being a reduction in the elongated neural morphology of the U251 N WT cells.

### Knock out of PAX6 increases colony formation and alter colony morphology

**Fig. 3:**
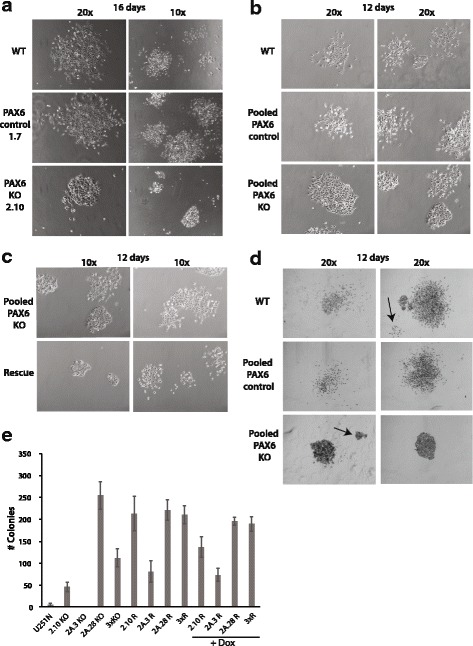
Knock out of PAX6 alters morphology of colonies and increases colony-formation ability. Five hundred cells (WT, Control or KO) per well in a 6-well plate was seeded and left for (**a**) 16 days or (**b**) 12 days to study colony forming abilities. **c** Five hundred cells of the Dox inducible EGFP-PAX6 pooled rescue cell lines and the pooled PAX6 KO cells were seeded per well in 6-well plates and left for 12 days. Colonies were micro-photographed. **d** Colonies were stained with 0.005% crystal violet before counting. Arrows indicate WT U251 N colonies containing less than 50 cells, and PAX6 KO colonies containing over 50 cells. **e** Five hundred cells were seeded in three wells in 6-well plates and left to form colonies for 12 days for each of the PAX6 KO cell lines (2.10 KO, 2A.3 KO and 2A.28 KO), and their Rescued counterparts (2.10 R, 2A.3 R and 2A.28 R) as well as the WT cell line U251 N. Pooled versions containing equal amounts of each of the three individual cell lines within each group were also included (3xKO, 3xR and 3xR + Dox). The term “+DOX” indicate Rescue cells treated with Dox 18–24 h before FACS sorting. The day after FACS sorting they were seeded for the colony formation assay. Rescue cells without Dox treatment were also included. Colony-forming abilities were measured by counting colonies consisting of over 50 cells. The figure is representative of three experiments done in triplicate. For each experiment the colonies were counted by two persons.

To investigate the cells ability to maintain cell growth and form colonies independent of contact with surrounding cells, we performed colony formation assays. It has previously been shown that mir-335 affect the colony forming abilities of glioma cells, and that regulation of PAX6 expression contribute to this [32]. We performed successful colony forming assays for the KO 2.10 cells, the PAX6 control 1.7 cells and the WT cells. The variety of morphology between the individual KO cell lines was reflected in the various forms of colonies observed in the assay. We observed three different types of colonies, tight, intermediate and loose, like described for U251 WT by Cao and colleagues [31]. Sixteen days after seeding, the WT cells had formed mainly large colonies. The colonies were loose or intermediate with a center of tightly packed cells. In addition, WT formed tight colonies after 12 days and 16 days (Fig. 3a, b, e). The PAX6 control 1.7 cells formed colonies similar to WT, while the PAX6 KO 2.10 cells formed mainly smaller tighter colonies, often with cells on top of each other or tightly packed (Fig. 3a). The PAX6 KO 2.10 cells covered a smaller surface area than the colonies formed by the WT- and the PAX6 control 1.7 cells, but they seemed to contain the same number (or even a higher number) of cells.

The three different PAX6 KO cells lines, and the four different PAX6 control cell lines, were pooled and used in colony-formation assays. The colonies formed by the pooled KO cells displayed more variety than what was observed for the 2.10 KO cells alone (Fig. 3b), probably reflecting the differences in morphologies observed for the individual KO cell lines (Fig. 3a). EGFP-PAX6 Rescue cells were also used in colony formation assays, and the colonies formed were smaller compared to the PAX6 KO cells, but had the same morphologies of tight, intermediate and loose (Fig. 3c). The colonies formed by PAX6 KO cells were denser and more tightly packed than the colonies formed by the WT and the PAX6 control cells, and thus strongly stained by crystal violet (Fig. 3d). Colonies were counted if they contained more than fifty cells. However, due to the tight packing of the colonies formed by the PAX6 KO cells, some colonies having more than fifty cells may have been overlooked during counting. As an illustration, arrows in Fig. 3d indicate PAX6 KO colonies estimated to have more than fifty cells, and WT colonies indicated to have less than fifty cells. Initial experiments clearly showed that KO cells had increased colony formation capabilities compared to the wild type U251 N cell line. Further, experiments with pooled Rescue cells indicated reduced colony formation compared to pooled KO cells after Dox stimulation, but the standard deviations were too large to be considered statistical relevant (data not shown). To clarify the colony forming ability of the individual cell lines, the three KO cell lines (2.10 KO, 2A.3 KO and 2A.28 KO) and the Rescue cell lines derived from these (2.10 R, 2A.3 R and 2A.28 R) were used in colony formation assays together with WT U251 N cells. For the Rescue cells, both Dox stimulated and unstimulated cells were used. The Dox stimulated Rescue cells were FACS sorted for EGFP-PAX6 expression 16–24 h after stimulation and allowed to recover for 1 day before they were seeded for this assay. The KO cell lines and the U251 N cell line were also grown in media containing Dox, so that potential differences observed are the result of induced PAX6 expression and not the presence of Dox. The experiment was done three times in triplicate, and a representative result is shown in Fig. 3e. This shows that the different KO clones have huge variations in their capability to form colonies, with the 2A.3 KO cell line being equally bad as the U251 N cell line in colony formation (less than 1% of seeded cells formed colonies), while the 2.10 KO and 2A.28 KO cell lines were far better, with efficiencies ranging from 7 to 22% for 2.10 KO and 32–51%for the 2A.28 KO in three experiments (Table 4). The ability of the Rescue cells to generate colonies were similar to their original KO counterpart, but especially the 2A.3 R cell line was better than its KO counterpart in colony formation. However, Dox stimulation and FACS sorting of EGFP-PAX6 expressing Rescue cells did not lead to changes in the ability to form colonies in any of the three Rescue cell lines (Fig. 3e).

**Table 4:** Percentage of colony formation ability in various PAX6 KO and Rescue cell lines

Cell lines	Exp.1	Exp.2	Exp.3
U251 N	0.3	0.6	1.0
2.10 KO	7.3	22.6	9.3
2A.3 KO	0.1	0.2	0.0
2A.28 KO	45.2	32.0	50.9
3xKO	21.4	17.2	22.4
2.10 R	7.0	40.8	42.7
2A.3 R	1.0	11.9	16.2
2A.28 R	20.3	42.1	44.2
3xR	19.5	34.1	42.1
2.10 R + DOX	17.1	35.5	27.4
2A.3 R + DOX	1.1	1.9	14.7
2A.28 R + DOX	19.8	37.6	39.1
3xR + DOX	19.4	28.9	38.0

To summarize, we have shown that PAX6 influences colony morphology, and that the ability to form colonies is enhanced in two of three PAX6 KO cell lines compared to the U251 N cells.

### Migration pattern and speed is altered by PAX6 KO

**Fig. 4:**
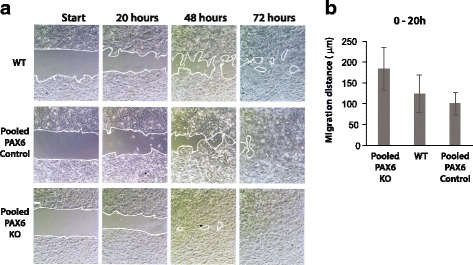
PAX6 KO cells migrate faster than WT and control cells. **a** WT U251 N cells, pooled PAX6 control cells and pooled PAX6 KO cells were seeded in Ibdi 2-well silicone inserts and left for 24 h. Cells were Mitomycin C treated for 2 h before inserts were removed, creating a cell free area (gap) at the start of the assay. Cells were micro-photographed at 0, 20, 48, and 72 h after well inserts were removed. The white lines demark migration edges. **b** The average migration distance was calculated after 20 h as described in methods. Students t-test showed that the difference in migration distance observed between pooled PAX6 KO cells and WT U251 N was significant (*p* < 0.002). The difference between pooled PAX6 Control cells and WT U251 N cells was not. Migration assays were repeated three times. Each assay showed the same result

The observed morphology of individual cells and cell colonies could indicate differences in mobility between the PAX6 KO cells and the PAX6 control (or WT) cells. To test this, migration assays were performed on pooled cells. The cells were treated with Mitomycin C to abolish proliferation. At 20 and 48 h after removal of well inserts, the PAX6 KO cells covered a larger area than WT and PAX6 control cells did (Fig. 4a). After 72 h, the entire region (originally cell-free at 0 h) was covered by the PAX6 KO cells. The WT and the control cells did not migrate at the same speed and there were still cell-free areas at 72 h after removal of well inserts. The differences in migration distance between the three cell lines are shown in Fig. 4b. These results are statistically relevant and show that the PAX6 KO cells have increased migration capacity compared to the PAX6 expressing cells; even though the morphology of WT cells (elongated, forming loose colonies) indicated that they would be more mobile. The results are in line with what others have found for PAX6 with regard to migration and invasion, e.g. in murine astrocytes reported by Sakurai et al. [33], and in glioblastoma cell lines, shown by Pavlakis et al. [34], Cheng et al. [32] and Mayes et al. [20].

### PAX6 KO cells have higher proliferation rate and altered cell cycle distribution compared to WT and PAX6 control cells

**Fig. 5:**
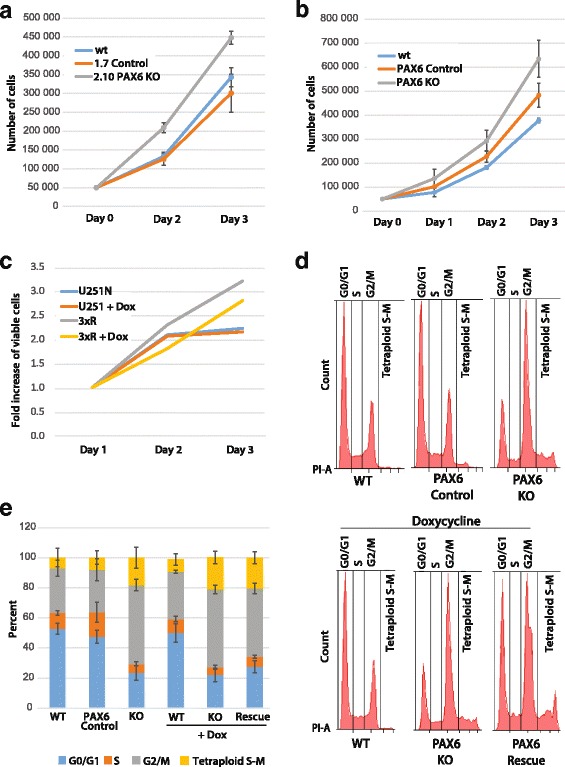
PAX6 restricts cell proliferation and regulates cell cycle. **a** Fifty thousand WT U251 N cells, PAX6 1.7 Control cells and PAX6 2.10 KO cells were seeded at day 0 and counted at day 2, and 3. Proliferation assays were repeated three times. **b** Fifty thousand WT U251 N cells, pooled PAX6 Control cells and pooled PAX6 KO cells were seeded at day 0 and counted at day 1, 2, and 3. **c** The Cell Titer Glo assay was used to compare proliferation (viability) in the WT U251 N cell line with the Rescue cell line treated with and without Dox. Dox treated Rescue cells were FACS sorted so that all cells expressed EGFP-PAX6. Values obtained at day 1 was set to 1, and the fold increase in proliferation at day 2 and 3 was calculated. Three independent experiments show similar results. **d** The cell cycle distributions of WT U251 N cells, pooled PAX6 control cells and pooled PAX6 KO cells at 70–90% confluency were examined by PI staining and FACS (upper panel). WT U251 N cells, pooled PAX6 KO cells and pooled PAX6 Rescue cells were Dox treated, and the cell cycle distributions were investigated at 70–90% confluency by PI staining and FACS (lower panel). **e** Average cell-cycle distribution from three experiments of cells mentioned in D. Student’s t-test, *p* < 0.05

In order to investigate the proliferation rate of the PAX6 KO cells compared to the WT cells, proliferation assays were performed by seeding cells and counting them at different time points. Regulation of PAX6 expression levels by miR-335 indicates that PAX6 restrict cell proliferation in glioblastoma cell lines [32]. The PAX6 KO 2.10 cells clearly proliferated faster than WT cells and the PAX6 control 1.7 cells (Fig. 5a). Pooled cells displayed the same pattern (Fig. 5b). The reintroduction of EGFP-PAX6 into the KO cells (generating Rescue cells) caused a clear reduction in proliferation when Dox stimulated and FACS sorted before use (Fig. 5c). PAX6 has been shown to be involved in cell cycle regulation in many cells and tissues, however it affects cell cycle differently in various cell types [17, 35–37]. We therefore wanted to investigate if there were changes in cell-cycle distribution in the PAX6 KO cells compared to the PAX6 expressing cells. The results showed that by knocking out PAX6 the percentage of cells in G0/G1 decreased more than 50% (Fig. 5d and e). This indicates that PAX6 keeps cells in the G0/G1 phase of the cell cycle. PAX6 KO cells showed a strong increase of cells in the G2/M phase (50% increase compared to the WT and the PAX6 control cells), with a strong reduction of cells in G0/G1- and S phase. For the PAX6 Rescue cells, the number of cells in the G0/G1 phase increased, while the number of cells in the G2/M phase decreased (Fig. 5d and e), creating a slight shift in the cell-cycle distribution for the PAX6 KO Rescue cells towards the WT distribution. To investigate if prolonged incubation with Dox would result in increased PAX6 expression (and thus less heterozygosity), the incubation time and concentration with Dox was increased to 6 days and 25 or 50 ng/ml. However, the results maintained the same. The difference in number of cells in G1/G0-phase for PAX6 KO cells compared to Rescue was small but significant, with a *p*-value < 0.05. Use of Dox stimulated and FACS sorted Rescue cells did not change this result (results not shown). Cyclin D1 and p27 have both been shown to be involved in PAX6 regulation of cell cycle in other cell lines [37, 38]. We therefore compared the expression of these genes in the PAX6 KO cells and WT cells, by use of RT-qPCR. We observed minor fold changes (− 1.5) between PAX6 KO and WT cells with regard to p27 expression. However, for cyclin D1 there was a fold change of − 2.4 in the PAX6 KO cells compared to the WT cells (Table 4). If cell cycle regulation by PAX6 was mediated by regulation of cyclin D1 expression, the opposite would be expected, that is; lower level of cyclin D1 in the WT cells that were arrested in the G1/G0 phase of the cell cycle. An alternative explanation for the low level of Cyclin D1 in the PAX6 KO cells is the fact that a higher proportion of these cells are in the G2 phase of the cell cycle, and thus would be expected to have lower level of Cyclin D1 expression since the expression varies through the cell cycle. Hence, by harvesting and comparing mRNA from cell populations with striking differences in their G1 to G2 phase distribution one would expect differences in the level of Cyclin D1 mRNA.

To summarize, PAX6 KO cells proliferate at a higher rate, and display altered cell cycle distribution with accumulation in the G2/M phase compared to the WT and PAX6 control cells.

### PAX6 KO cells display enhanced resistance to oxidative stress

There are reports linking PAX6 to oxidative stress in glioblastoma cells [23]. H_2_O_2_ is naturally produced in large amounts by tumor cells [39–42]. To assess whether PAX6 KO, WT and control cells were differently affected by oxidative stress we exposed the PAX6 2.10 KO cells, the WT and the PAX6 1.7 control cells to various concentrations of H_2_O_2_ for 24 h. At the H_2_O_2_ concentration of 60 μM the PAX6 2.10 KO cells displayed minor changes, with a small proportion of the cells rounded up. However most of the cells still looked vital and attached. Similareffect was obtained using a 3 times lower concentration of H_2_O_2_ (20 μM) on the WT and the PAX6 1.7 control cells. At 60 μM H_2_O_2_ most of the WT and PAX6 1.7 control cells were rounded up or detached from the surface (data not shown). This confirmed that the presence of PAX6 renders the cells more sensitive to oxidative stress.

**Fig. 6:**
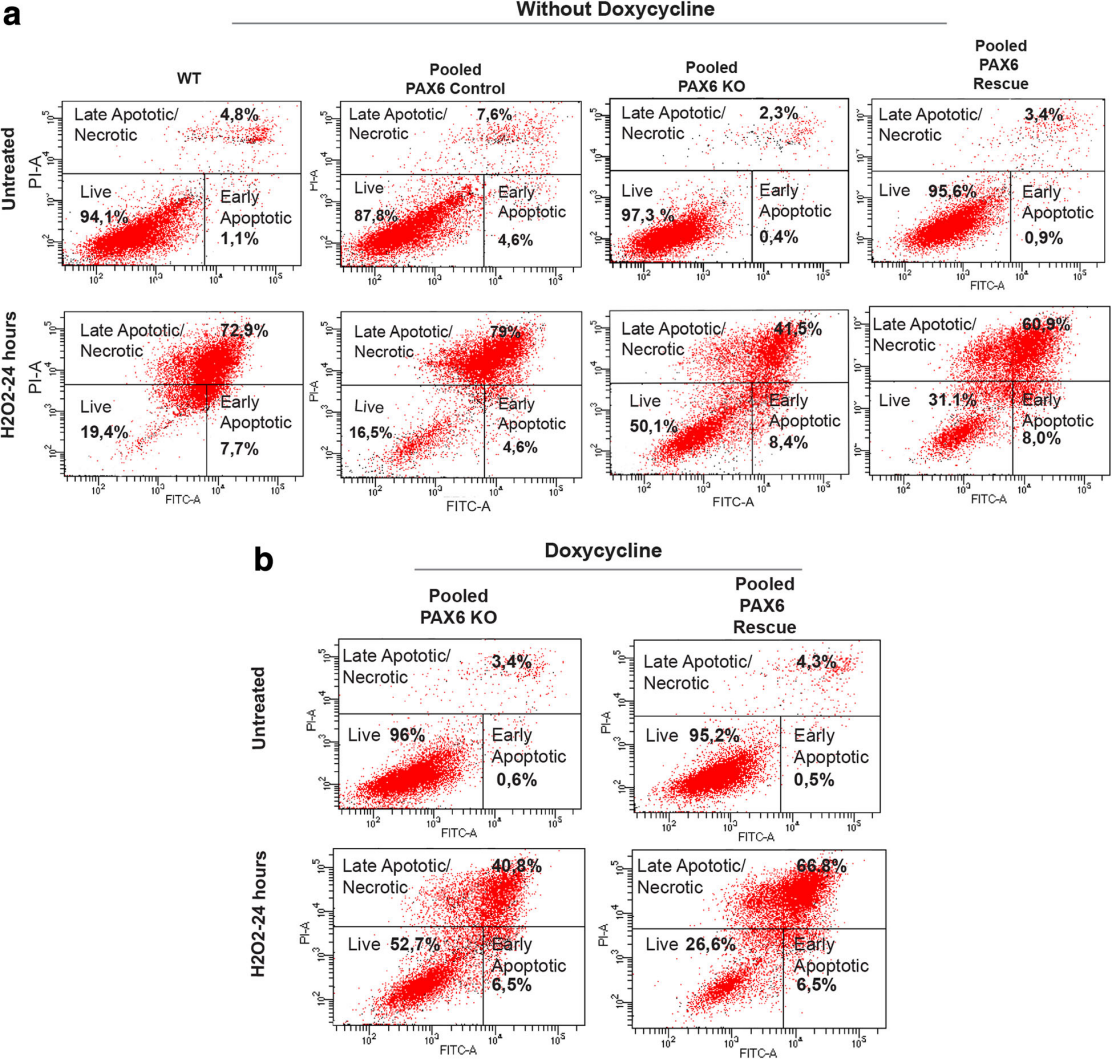
PAX6 KO cells are less sensitive to oxidative stress. Cells were exposed to 300 μM H_2_O_2_ for 24 h, and Annexin V FITC /PI stained before FACS analysis. Percentage of viable cells, cells in early apoptosis and cells in late apoptosis/necrosis were determined. **a** Cells without Doxycycline treatment. **b** Doxycycline treated cells. Figure shows representative results from three to four experiments

To investigate this further, we incubated pooled PAX6 KO cells, WT, PAX6 control and PAX6 Rescue cells in 300 μM H_2_O_2_ for 24 h. The concentration of 300 μM was chosen as Chang and colleges found that PAX6 increased glioma cell sensitivity to detachment induced oxidative stress at ROS levels comparable with what 300 μM H_2_O_2_ induce [23]. Cells were stained with PI and anti-Annexin V FITC, and analyzed by FACS. The results strongly support that the PAX6 KO cells have a higher tolerance to H_2_O_2_ induced oxidative stress than WT cells (Fig. 6a and b). While 50% of the H_2_O_2_ treated KO cells were healthy and intact, less than 20% of the WT and PAX6 control cells were viable. The highest percentage of late apoptotic and dead cells (79%) were found in the sample of PAX6 control cells, closely followed by the WT cells (72.9%). For PAX6 KO cells only 41.5% were found to be late apoptotic or dead. In line with this, the PAX6 Rescue cells displayed less viable cells and increased the percentage of apoptotic cells upon H_2_O_2_ treatment compared to the PAX6 KO cells (Fig. 6a). This was observed for the cells even without Dox treatment, and we believe that is due to the leakiness of the Dox inducible system. However, the difference was more pronounced for Dox induced cells (Fig. 6b). While Dox in combination with H_2_O_2_ had no effect on the amount of live PAX6 KO cells (52.7% vs 50.1% in Dox stimulated vs unstimulated cells), the combination of Dox and H_2_O_2_ caused even more reduction in live Rescue cells (26.6% vs 31.1% in Dox stimulated vs unstimulated cells) (compare respective panels in Fig. 6a and b). This indicates that the increased PAX6 expression in the cells make them more sensitive to H_2_O_2_. To summarize, these results show that depletion of PAX6 affects cell morphology, cell proliferation, cell cycle distribution and sensitivity to oxidative stress – and these changes are reversed by re-introduction of PAX6 using a Dox inducible PAX6 expression vector. Furthermore, PAX6 depletion also highly enhanced the formation of colonies in a colony formation assay. However, this was not reversed by reintroduction of PAX6.

### TMZ is more lethal to PAX6 KO cells compared to WT and control cells, while the opposite is observed for Withafarin a

**Fig. 7:**
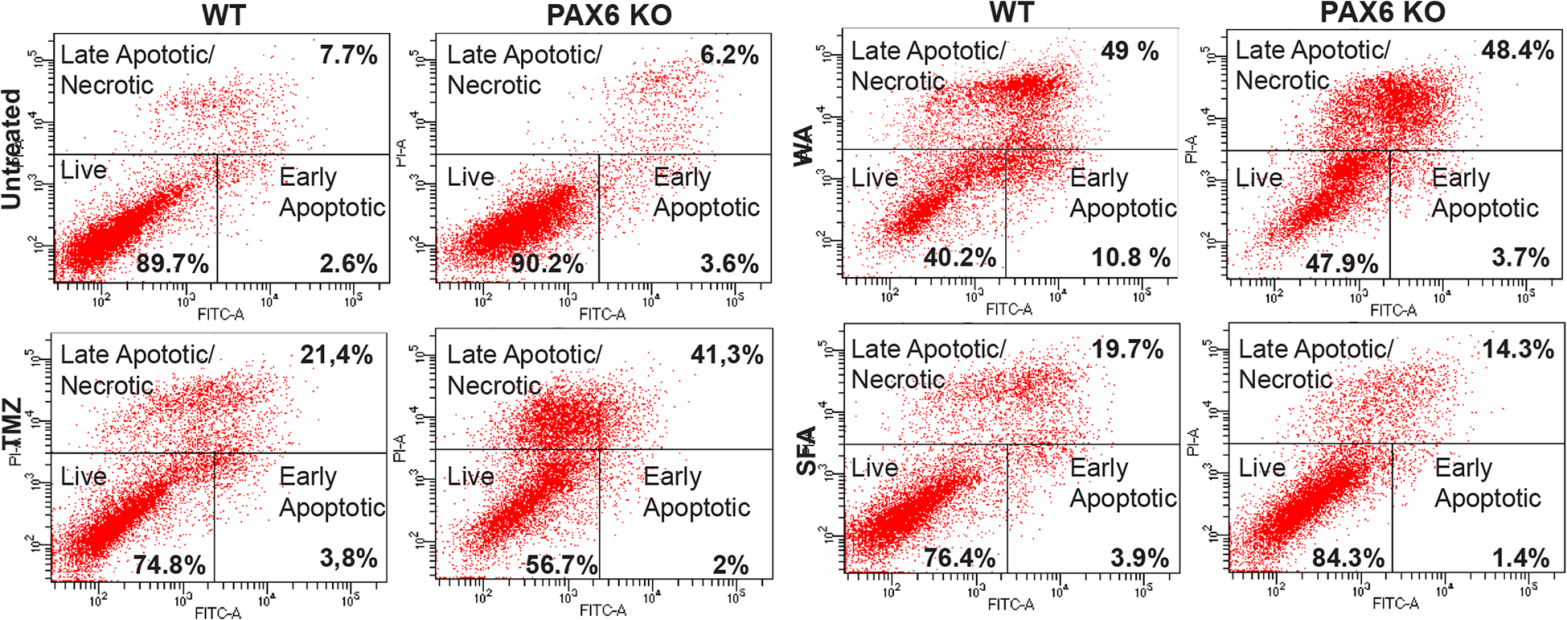
PAX6 KO cells respond differently to treatment with the chemotherapeutic drugs TMZ, WA and SFA compared to WT cells. U251 N PAX6 KO and WT cells were treated with 250 μM Temozolomide (TMZ), 1.5 μM Withaferin A (WA) and 10 μM Sulforophane (SFA) for 48 (WA and SFA) or 72 (TMZ) hours and analyzed for apoptosis by anti-Annexin/PI staining and FACS. The experiment shown is representative of three independent experiments

To investigate if PAX6 and its ability to render cells more sensitive to oxidative stress would play a role in treatment of glioblastoma, we treated the PAX6 KO cells and controls with various chemotherapeutic agents; Temozolomide (TMZ), Withaferin A (WA) and Sulforaphane (SFN). TMZ treatment, 250 μM for 72 h, reduced the cell number of both the WT and the PAX6 KO cells compared to untreated cells as observed in the microscopy. FACS was used to investigate the proportion of live and dead cells. As shown in Fig. 7, the TMZ treated PAX6 KO cells had more than double the amount of cells in the category late apoptotic/necrotic/dead cells compared to the WT cells. This clearly shows that PAX6 expression is beneficial for cell survival after TMZ treatment. WA treatment, 1.5 μM for 48 h, showed no differences in the percentage of cells in late apoptosis/necrosis for the WT (49%) and KO (48.4%). However, there were less live cells, and more early apoptotic cells for the WT (40.2 and 10.8% respectively) than for the PAX6 KO cells, where the comparable numbers for live and early apoptotic cells were 47.9 and 3.7%, respectively (Fig. 7). WA induces oxidative stress in cancer cells [43] and that may be the reason for WT cells being more sensitive to the treatment compared to PAX6 KO cells. Treatment with SFN, 10 μM for 48 h, seemed to strongly reduced proliferation in both PAX6 KO and control cells when observed in the microscopy. PAX6 KO cells displayed a slightly higher percentage of vital cells (84.3%) compared to the WT cells (76.4%), which had more apoptotic cells in the FACS analyses (Fig. 7). This strongly support that PAX6 expression sensitizes U251 N cells to chemotherapy treatment.

### PAX6 KO and WT cells show differences in gene expression for MMP2, CAV1, cyclin D1 and Nrf2

Changes in gene expression between PAX6 KO cells and WT cells were studied by RT-qPCRs for selected genes (MMP2, CAV1, Cyclin D1, p27, and Nrf2) (Table 4) known to be regulated by PAX6, or known to participate in proliferation, migration and/or redox regulation. MMP2 is involved in migration and is a known PAX6 target [20]. To our surprise, the PAX6 KO cells showed a 5-fold reduction of MMP2 compared to WT cells (Table 5). This is contradictory to what others have shown [20]. Another gene involved in migration and proliferation is CAV1. RT-qPCR showed a 2-fold downregulation of CAV1 in the PAX6 KO cells, which is in line with what we have observed when PAX6 was knocked down in the pancreatic adenocarcinoma cell line HPAFII (Forsdahl et al: in preparation). In the HPAFII cell line, knockdown of PAX6 caused enhanced proliferation and migration, in addition to the reduced expression of CAV1 (results not shown). Cyclin D1 and p27 are known target genes of PAX6 [44, 45], and important molecules regarding the regulation of cell cycle progression. PAX6 KO cells had a 2.4 fold downregulation of cyclin D1 compared to WT, while p27 expression was not significantly affected by PAX6 KO (Table 5). High levels of Cyclin D1 is required for the progression from G1 to S in the cell cycle. The expression of cyclin D1 is expected to be low or absent in most of the other cell cycle phases, although cancer cells may show aberrant regulation. A reduction in PAX6 KO cells in the G0/G1 phase compared to WT cells (as shown in Fig. 5D), might reflect the difference in cell cycle distribution between the PAX6 KO and WT cells, and not the PAX6 regulation of Cyclin D1 expression. Nfr2 is a key regulator of the oxidative stress defense mechanisms, and controls the basal and the induced expression of an array of antioxidant response element–dependent genes [46]. PAX6 KO cells has a 3.7 fold downregulation of Nrf2 compared to WT cells (Table 5), which is interesting since we have shown that these cells are in fact more resistant to oxidative stress by H_2_O_2_. PAX6 expressing cells are thus more sensitive to oxidative stress despite having higher expression of Nrf2 than the PAX6 KO cells. This urge that the effect of PAX6 on other regulators of oxidative stress responses should be included in further studies.

**Table 5:** RT qPCR shows negative fold change of gene expression in PAX6 KO cells compared to WT. ∆∆Ct method was used for analysis

Gene abbreviation	Protein name	Fold change # ± SD
*MMP2*	Matrix metalloproteinase 2	−5.0 ± 1.50
*CAV-1*	Caveolin-1	−2.0 ± 0.24
CCND1/ cyclin D1	Cyclin D1	−2.4 ± 0.14
*CDKN1B/ p27*	Cyclin-dependent kinase inhibitor 1B (p27^Kip1^)	−1.5
*NFE2L2 / Nrf2*	Nuclear factor (erythroid-derived2)-like 2 NFE2L2 or Nrf2	−3.7 ± 0.68

The relative amount of target gene normalized to the average expression of the two reference genes *TFRC* and *GAPDH* was determined using the ΔΔCt-method [71]. WT values were put as 1, and fold change according to this were calculated for the other cell types/treatments

## Discussion

PAX6 is frequently expressed in tumors [13–16]. In glioblastoma, PAX6 expression is associated with glioma grade. From the development of anaplastic astrocytoma into stage IV glioblastoma the expression level of PAX6 decreases by 3 fold, and GBM tumors have 2–12 fold less PAX6 expression compared to surrounding normal tissue [18].

In our studies, we have as the first created a tool by successfully knocking out PAX6 in the U251 N glioblastoma cell line using the CRISPR-Cas9 technology. We have demonstrated that complete absence of PAX6 by KO causes increased proliferation, migration and colony forming abilities, confirming that PAX6 acts as a tumor suppressor in glioblastoma cells lines. We also observed that one of the three morphology types normally present in U251 N cells dominates in the KO cells. The U251 N cells neuron-like elongated morphology was reduced. This was interesting, as PAX6 has been demonstrated to alter cell morphology of HeLa cells when introduced by transfection of a lentiviral vector. The HeLa cells formed neurite-like extensions, and neuron-specific genes were upregulated [47]. The dominant morphology of the colonies derived from single PAX6 KO cells was also different from the one observed with WT cells. Furthermore, a shift in cell cycle distribution was apparent in the KO cells where the majority of the cells was in G2/M-phase, while for WT cells the majority was in G0/G1. This suggests that PAX6 is involved in keeping the cells in the G0/G1 phase of the cell cycle. Rescuing the KO cells through viral transduction of an EGFP-PAX6 expressing vector, led to a slight reduction of cells in the G2/M-phase with a corresponding increase in number of cells in G0/G1. We also found that PAX6 KO cells are more resilient than WT cells to oxidative stress caused by exposure to H_2_O_2_. The rescue cells containing EGFP-PAX6 clearly made the cells more sensitive to oxidative stress.

We found a variety of CRISPR-Cas9 generated mutations in the PAX6 gene when genomic DNA from the knock out cell lines were cloned and sequenced (Additional file 2: Figure S2). Although U251 cells and other cancer cells have ongoing chromosomal- and chromosome structure instabilities [48] including variation in chromosome number [25], it is unclear whether chromosome 11, where PAX6 is located, is amplified [48, 49]. Regardless of this, the fact that we observe three to seven different mutated sequences for all our single cell clones either imply that they were not single cells upon sorting, or that one or both of the PAX6 alleles in the single cell were not mutated by CRISPR-Cas9 at the time of sorting, even though the cells expressed the vector marker EGFP. Mutations generated by the CRISPR-Cas9 construct after the first and second cell-division would then contribute to the observed variation in mutation sequences. Importantly, complete absence of PAX6 protein was confirmed by western blot and immunocytochemistry for all the generated PAX6 KO cells.

The generated PAX6-EGFP inducible rescue cells were able to rescue the observed effect of the PAX6 KO on proliferation and oxidative stress, but they were not able to shift the cell cycle distribution back to the one observed for the WT cells, nor were they able to reduce the colony forming abilities or the changes in gene expression for the selected genes. Since PAX6 is known to function in a dose dependent manner [50, 51] we initially thought that the partial and heterogenous expression of PAX6 upon Dox exposure (revealed by the ICC) was the main reason for this. Not all Rescue cells expressed EGFP-PAX6, and the ones that did, did it at different concentrations. The selection of EGFP-PAX6 expressing cells by FACS ensured that all cells in the experiment expressed PAX6, but the level of PAX6 expression in each cell was still variabel. The dependency on correct dose of PAX6 might vary from one biological process to another, and might explain why we observed a rescue with regard to e.g. proliferation and not colony formation. In addition, the time the cells were exposed to the PAX6 protein before the colony formation assay, cell cycle distribution experiments or gene expression studies were started, might play a role, depending on the target genes and pathways affected by PAX6. In theory, removal of PAX6 in the PAX6 KO cells may have started a change in the cells that are “locked”, and not reversed by just adding PAX6 back again (e.g chromatin modifications making PAX6 target genes inaccessible). Finally, the rescue construct provided the main isoform PAX6, and not the PAX6(5a) isoform, which is shown to play a part in eye, pancreas, neural development, pancreatic cancer and glioblastoma development [34, 52, 53]. Recently, PAX6(5a) was shown to play an important role in regulation of glioma progression by reducing cell survival and decreasing migration and invasion in glioblastoma cell lines [34]. It did this by downregulating the lipid kinase SPHK1, a gene previously identified by our research group as a PAX6(5a) specific target gene [27].

U251 N WT cells have three different morphologies of cells and colonies derived from single cells [31]. We observed that removal of PAX6 made the small cobblestone morphology dominant. This morphology forms tight colonies. However, the two other morphology types were also observed, but to a lesser extent than what was observed for WT cells. It has previously been shown that PAX6 is involved in regulating cell morphology both in cerebellar granule cells during neurogenesis and in pancreas [52, 54, 55]. Yamasaki et al. [54] found that PAX6 mutant (sey^2^/sey^2^) cerebellar granule cells are less elongated than WT cells and have random migration that may be due to aberrant cytoskeletal control. The majority of the PAX6 KO cells in our experiments are also less elongated. Interestingly, we observed that the PAX6 KO cells migrate mainly as sheets, while WT and PAX6 control have more elongated cells extending out in the direction it is heading. This indicates that our PAX6 KO cells have cytoskeletal changes that not only affect the morphology, but also the pattern of migration.

The PAX6 KO cells migrated more efficiently than the WT cells in scratch assays. Changes in speed of cell migration by PAX6 KO or knockdown is also shown by others [52, 56, 57]. Astrocytes from PAX6 mutant (sey/sey) mice are shown to migrate faster than astrocytes from WT mice in scratch assays [33]. It has also been shown that U251 cells that stably overexpress PAX6 show reduced migration and invasion capacity [20].

Several publications show, that PAX6 affects cell proliferation, but the effect is cell-, tissue- and context dependent [17, 38, 52, 58]. We found that absence of PAX6 in the U251 KO cells increased both the proliferation and the ability to form colonies. This is in line with what others have found. Colony formation is an in vitro cell survival assay based on the ability of a single cell to grow into a colony. The size of the colony is affected by the cells doubling time. Proliferation and colony formation of glioblastoma cells increased when miR-335 caused an approximately 2-fold downregulation of PAX6 [32], and astrocytes from PAX6 (sey/sey) mutant mice proliferate faster than the corresponding cells in the WT mice [33]. Others have shown that over-expression of PAX6 in U251 cells reduced colony formation ability in soft agar assays, but it did not affect cell doubling time in U251 [17]. Complete absence of PAX6 in our KO cells increased colony formation, and by that we confirmed the role of PAX6 in reducing the glioblastoma cell lines ability to form colonies from a single cell.

PAX6 is reported to influence the cell cycle distribution in different cell types, however the mechanisms and direction of regulation are cell context- and tissue- dependent [17, 35, 38, 58]. In our study, we retrieved clear results showing that the percentage of PAX6 KO cells in the G0/G1 phase of the cell cycle decreased, while the percentage found in the G2/M phase increased compared to WT cells. This is in line with what others have observed for glioblastoma cells [17] and corneal epithelial cells [58]. We found that reintroducing PAX6-EGFP to our KO cells increased G1/G0 and lowered G2/M-phase. However, the percentage of PAX6 rescue cells in the G0/G1 phase did not reach the same level as for WT. With the possible dose-dependency of PAX6 in mind, we increased Dox concentration to elevate PAX6 expression. The cells were incubated in Dox for up to 5 days, but cell cycle distribution remained the same as for 24 h Dox stimulation (data not shown). FACS sorting of cells after Dox induction gave the same result. In glioblastoma cell lines and in corneal epithelial cells, *PAX6* overexpression retains cells in G0/G1, retarding the passage of cells through the cell cycle [17, 58]. The study of the corneal epithelial cells was conducted using a Dox-inducible system in WT cells, as we did in our rescue cells. While our rescue cells were a heterozygous mix of different expression levels. Their inducible cells were selected for high homozygous expression of PAX6 after 24 h of Dox induction, which was the time frame of Dox-induction during their experiments. By use of adenoviral transductions of U251 to overexpress PAX6, or the use of a dominant negative PAX6, Zhou and colleagues [17], concluded that PAX6 suppresses cell growth by inhibition of the G1/S transition. Interestingly, although PAX6 was shown to inhibit cell proliferation also in HeLa cells, the accumulation of cells was in the G2/M phase [59]. Our studies support that PAX6 has a role in G1/S cell cycle arrest, since we see a shift in the cell cycle distribution from G0/G1 in WT cells to G2/M in PAX6 KO cells. Although it seems that most studies support an inhibitory role for PAX6 with regard to cell cycle regulation, it is reported that in in lung cancer PAX6 promote cell cycle transition from G1 to S phase [38].

PAX6 has been shown to exert its effects on cell cycle progression by various mechanisms. In HeLa cells PAX6 has an antiproliferative effect by increasing expression of human RFPL1 (Ret finger protein-like 1) that controls cell cycle progression through cyclin B1/Cdc2 degradation. This delays mitosis entry and causes accumulation of cells in G2/M-phase [59]. In lung cancer PAX6 knockdown cell lines, cyclin D1 is suppressed, indicating that PAX6 promote cell cycle transition from G1 to S-phase [38]. In addition, the pRB (Retinoblastoma) S780 phosphorylation level is decreased in lung cancer PAX6 KD cells. Cyclin D complexes phosphorylate the pRB family of nuclear phosphoproteins to regulate the G1/S transition [60]. The PAX6 regulation of cell cycle in lung cancer cells was through the MAPK signal pathway as they found that reduction of PAX6 decreased ERK1/2 and p38 phosphorylation [38].

Cancer cells produce high levels of reactive oxygen species (ROS), and are under intrinsic oxidative stress [39]. In the increased necrotic areas that are one of the characteristics of GBM, the level of ROS is elevated [3]. Cells must be resilient to the oxidative stress to maintain viability. Interestingly, we found that the PAX6 KO cells were substantially more resistant to oxidative stress generated by addition of H_2_O_2_, compared to PAX6 WT cells. When incubated in 300 μM H_2_O_2_ for 24 h we observed that 48% of PAX6 KO cells versus 74% PAX6 WT cells were in late apoptosis. We also found that KO cells had the morphology of healthy and viable cells at H_2_O_2_ concentrations that induced WT cells to round up, detach and enter apoptosis. PAX6 Rescue cells were more sensitive to oxidative stress than the PAX6 KO cells. Chang et al. [23] found that detachment of U251 cells by trypsin or scraping increased ROS levels, and that this made U251 cells that over-expressed PAX6 more susceptible to detachment-induced stress compared to WT U251. One knows very little about the mechanisms that render PAX6 positive cells more sensitive to increased ROS levels, but our results confirm the finding of Chang and colleagues and show that several sources of ROS have the same effect on glioma cells expressing PAX6 [23].

Temozolomide (TMZ) is an alkylating agent which delivers a methyl group to purine bases in DNA [61]. TMZ is used as standard treatment for high-grade gliomas [61]. GBM cells acquire chemo resistance to this drug as well as to ionizing radiation and other chemotherapeutic agents that elevate intracellular ROS [31]. TMZ causes increased PAX6 expression in glioblastoma cell lines U251 and U118, and TMZ is reported to depend on PAX6 expression to decrease proliferation of GBM cells [24]. The oxidative cytotoxic agent Withaferin A (WA) is a candidate for GBM treatment. It re-sensitizes TMZ-resistant gliomas [62]. Grogan and colleagues found that this re-sensitization works through depletion of O6-methylguanine-DNA methyltransferase (MGMT) and the induction of apoptosis through the AKT/mTOR pathway [62]. Knowing that WA increases oxidative stress [62] and that TMZ increases PAX6 expression (which sensitizes cells to oxidative stress), can possibly provide an explanation of the molecular mechanisms behind the combined effect of WA and TMZ. In TMZ treated cells, we did not observe any substantial differences in proliferation inhibition between the WT and PAX6 KO cells by simple microphotography, though the PAX6 KO cells proliferated faster than WT as in untreated cells. However, in FACS analyses we observed a doubling in percentage of cell death induced by TMZ in PAX6 KO cells compared to WT cells (41.3% vs 21.4%). Hence, it is clear that knocking out PAX6 renders the cells more sensitive to apoptosis from TMZ treatment. The opposite was observed with WA, as this drug reduced viability in the WT cells to a greater extent then what was observed for PAX6 KO cells. The increased effectiveness of WA treatment in WT cells is likely because of the drugs ability to induce oxidative stress, which PAX6 expressing cells are more sensitive to. Sulforaphane (SFN) was efficient in reducing proliferation in both PAX6 KO and WT cells, however PAX6 KO cells had a higher percentage of viable cells compared to WT in FACS assays. SFN is known to induce growth inhibition and apoptosis in human GBM cell lines GBM 8401 and U251 [63, 64], and our results indicate that removal of PAX6 makes the cells less sensitive to this drug. SFN can similar to WA, reverse TMZ-resistance in glioblastoma cells. SFN downregulates MGMT expression by inhibiting the NF-κB pathway [65].

RT-qPCRs were performed to study the effect of PAX6 on various genes in U251 N. PAX6 KO cells had a reduction of MMP2 at − 5 fold compared to WT. MMP2 is involved in migration and is a known PAX6 target, and others have found PAX6 to suppress MMP2 expression [20]. This is the contrary to what we show in our KO cells; however, Mayes and colleges studied cells overexpressing PAX6, and they used a different U251 cell line. CAV1 is another gene involved in migration and proliferation. We have identified CAV1 as a positively regulated target gene of PAX6 in human pancreatic adenocarcinoma cell line HPAFII (Forsdahl et al: in preparation). CAV1 is known to reduce proliferation and clonogenicity of U87MG GBM cells, and downregulation of CAV1 increase the cells proliferation ability and invasiveness [66]. We found that CAV1 is 2-fold downregulated in the PAX6 KO cells compared to WT, which fit with observations in other glioblastoma cell lines where lower expression of CAV1 equals increased proliferation. Since cell cycle distribution was altered in the PAX6 KO cells, RT-qPCRs on cyclin D1 and p27 were performed. PAX6 regulates Cyclin D1 in lens cells and p27 in neuroepithelial (NE) progenitor cells in the mouse optic vesicle [44, 45]. We did not find significant changes in p27 expression when PAX6 was absent. However, the PAX6 KO cells had a 2.4 fold downregulation of cyclin D1 compared to WT cells. Cyclin D1 is important for progression through the G1/S checkpoint [67], and is normally high in G1/M phase, reduced in S-phase and then elevated again in G2 phase to promote proliferation [68, 69]. In addition, shRNA knockdown of Cyclin D1 has previously been shown to induce apoptosis and inhibit proliferation of U251 cells [70]. However, since we observe increased proliferation in the PAX6 KO cells despite the downregulation of Cyclin D1, there must be other factors responsible for the increased progression through cell cycle and increased proliferation in the PAX6 KO cells compared to WT. We observed that PAX6 KO renders U251 N cells less sensitive to oxidative stress. The TF nuclear factor erythroid 2 (NFE2)-related factor 2 (Nrf2) is a key regulator of genes involved in oxidative stress defense [46]. We found the Nrf2 expression to be regulated by PAX6 in glioblastoma cells, although it cannot be responsible for the increased resistance for oxidative stress observed in our PAX6 KO cells, since it is downregulated by 3.7 fold in this cell line. The RT-qPCR results differing from theories based on what others have found shows the complexity of the processes involved in increased tumorigenecity in the PAX6 KO cells and in glioblastomas in general.

## Conclusion

With our novel tool and new approach to study the role of PAX6 in glioblastoma, we have established that PAX6 influence the cell cycle distribution, and renders U251 cells more sensitive to oxidative stress. Importantly, we discovered differences in the sensitivity to established chemotherapeutic drugs between the PAX6 expressing (WT) cells and the PAX6 KO cells. The PAX6 KO cells are less sensitive to oxidative stress, and to the drugs WA and SFN, compared to WT cells. However, for TMZ treatment PAX6 KO cells show more sensitivity and cell death than WT cells. Since the level of the PAX6 protein expression changes with the grade of glioblastoma, the observed variation in drug responses can be valuable information when planning treatment and developing new chemotherapies for GBM patients. It will therefore be interesting to further study the mechanisms behind the variation in cell responses to WA and TMZ treatment for the PAX6 KO cells and WT cells.

## Additional files

Additional file 1: **Figure S1.** Localization in the PAX6 gene of the three guide RNAs for CRISPR-Cas9 editing. Guide RNAs are located downstream of the translational start site (exon 1) for generating frameshift mutations and introduce STOP codons in the 5′-end of the PAX6 gene. Sequencing results of PAX6 knock out clones are included in supplementary. (TIF 4289 kb)

Additional file 2: **Figure S2.** Sequencing of the PAX6 knock out cells showed various mutations, deletions and insertion leading to STOP codons in the N-terminal of the PAX6 protein. Number of different sequences from the PAX6 knock out clones**;** (A) Seven sequences from clone 2A.3. (B) Seven sequences from clone 2A.28. (C) Six sequences from clone 2.10. (PDF 2920 kb)

## Abbreviations

Dox: Doxycycline
GBM: Glioblastoma
ICC: Immunocytochemistry
KO: PAX6 knock out
ON: Overnight
RT: Room temperature
SFN: Sulforaphane
TMZ: Temozolomide
TSS: Transcription start site
WA: Withaferin A
WT: Wild Type
