# Supplementary figures and images for: Generation of a PAX6 knockout glioblastoma cell line with changes in cell cycle distribution and sensitivity to oxidative stress
Source: BMC Cancer. 2018 May 2;18:496. doi: 10.1186/s12885-018-4394-6 (PMC5930953; doi:10.1186/s12885-018-4394-6)

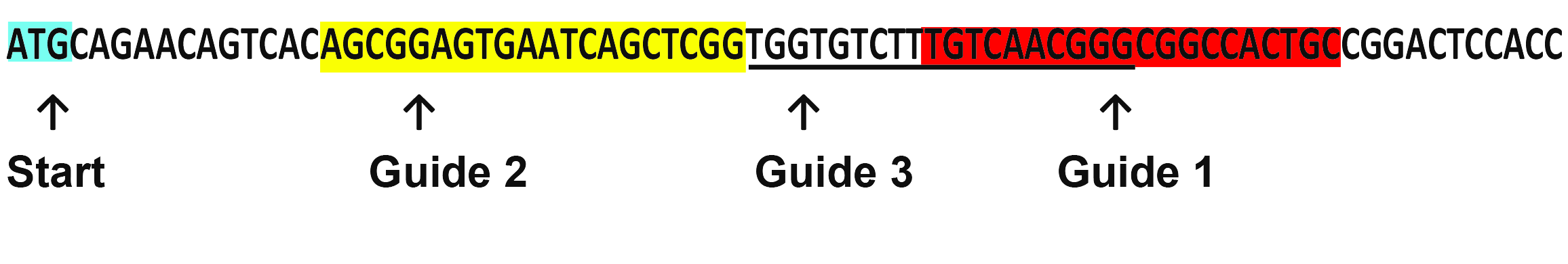

Supplement: Supplementary file 1 — Figure S1. Localization in the PAX6 gene of the three guide RNAs for CRISPR-Cas9 editing. Guide RNAs are located downstream of the translational start site (exon 1) for generating frameshift mutations and introduce STOP codons in the 5′-end of the PAX6 gene. Sequencing results of PAX6 knock out clones are included in supplementary. (TIF 4289 kb) [file 12885_2018_4394_MOESM1_ESM.tif]

**Figure 2****A**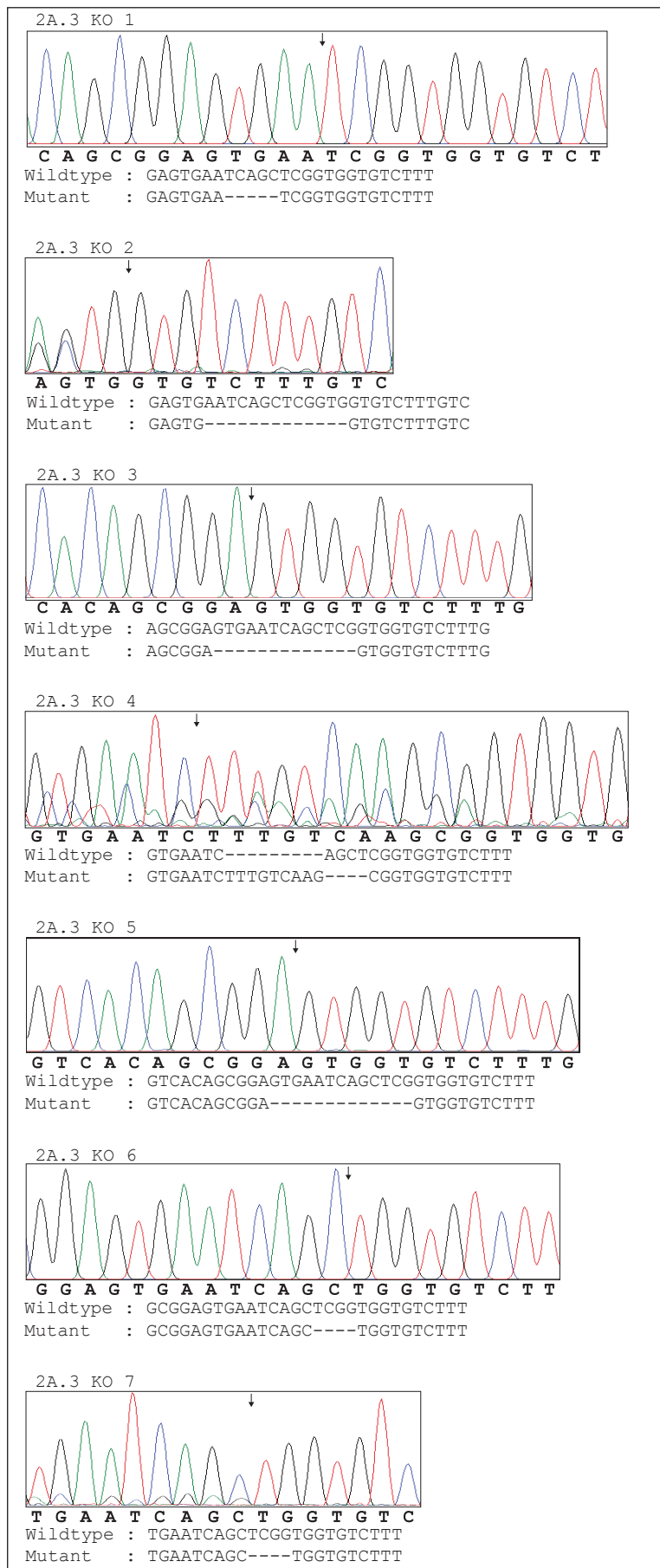**B**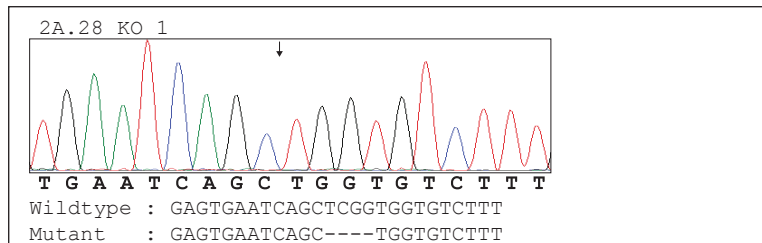

**B**

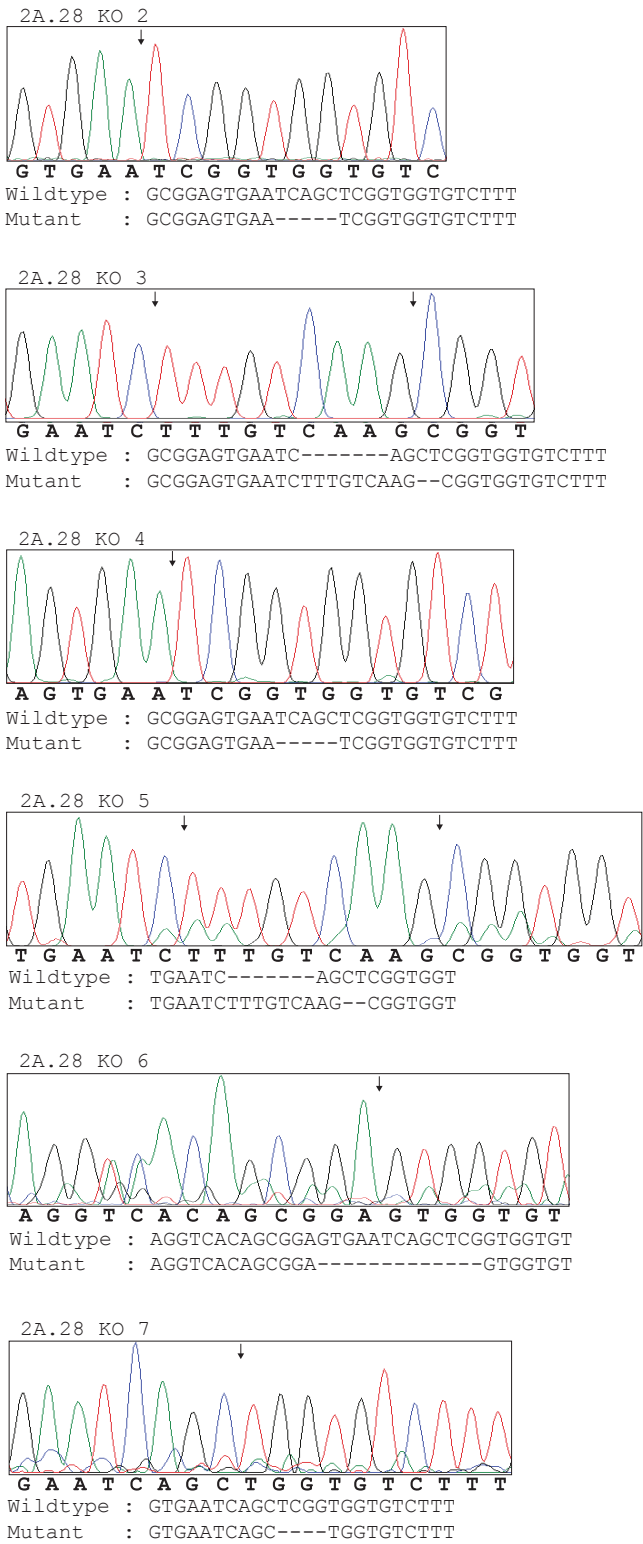

**C**

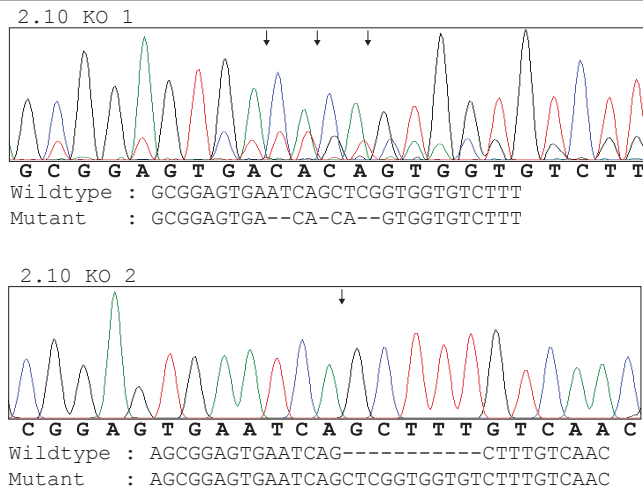

**C**

2.10 KO 3

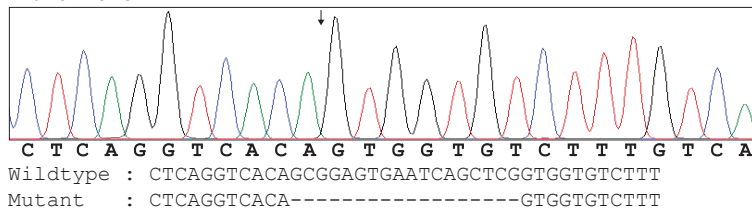

2.10 KO 4

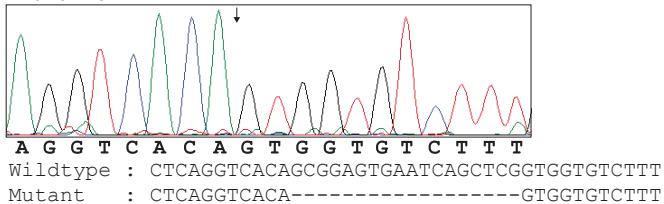

2.10 KO 5

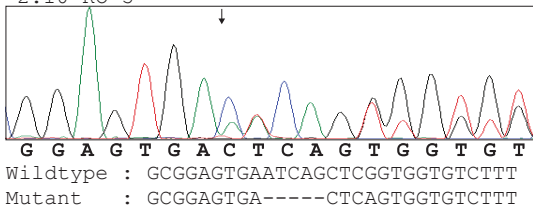

2.10 KO 6

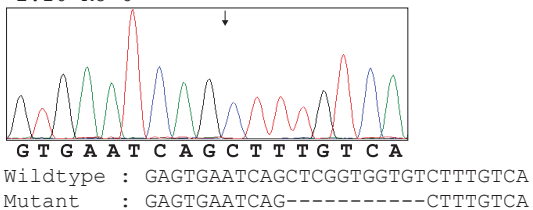

Supplement: Supplementary file 2 — Figure S2. Sequencing of the PAX6 knock out cells showed various mutations, deletions and insertion leading to STOP codons in the N-terminal of the PAX6 protein. Number of different sequences from the PAX6 knock out clones; (A) Seven sequences from clone 2A.3. (B) Seven sequences from clone 2A.28. (C) Six sequences from clone 2.10. (PDF 2920 kb) [file 12885_2018_4394_MOESM2_ESM.pdf]
